# The Association Between Statin Drugs and Rhabdomyolysis: An Analysis of FDA Adverse Event Reporting System (FAERS) Data and Transcriptomic Profiles

**DOI:** 10.3390/genes16030248

**Published:** 2025-02-21

**Authors:** Robert Morris, Kun Bu, Weiru Han, Savanah Wood, Paola M. Hernandez Velez, Jacob Ward, Ariana Crescitelli, Madison Martin, Feng Cheng

**Affiliations:** 1 Department of Pharmaceutical Science, Taneja College of Pharmacy, University of South Florida, Tampa, FL 33613, USA; rpm4@usf.edu (R.M.); savanahwood@usf.edu (S.W.); hernandezvelez@usf.edu (P.M.H.V.); jacobward@usf.edu (J.W.); crescitelli@usf.edu (A.C.); madison50@usf.edu (M.M.); 2 Department of Mathematics & Statistics, College of Art and Science, University of South Florida, Tampa, FL 33620, USA; kunbu@usf.edu (K.B.); weiruhan@usf.edu (W.H.)

**Keywords:** FAERS, pharmacovigilance, drug interactions, muscle disease, RNA-seq, statins, rhabdomyolysis, association rules, kidney injury, DDI

## Abstract

Background/Objectives: Rhabdomyolysis, a dangerous breakdown of skeletal muscle, has been reported as an adverse event in those prescribed a statin therapy for the treatment of hypercholesterolemia. Statin drugs are some of the most prescribed treatments for elevated cholesterol levels. The purpose of this comparative study was to determine the association between the statin drugs used and the risk of rhabdomyolysis using the FDA Adverse Event Reporting System (FAERS) and transcriptomic data. Methods: A disproportionality analysis was performed to compare the risk of rhabdomyolysis between the reference statin drug (simvastatin) and the treatment group, with patient age assessed as a possible confounder. In addition, association rule mining was utilized to both identify other adverse events that frequently presented with rhabdomyolysis and identify possible drug-drug interactions (DDIs). Finally, public transcriptomic data were explored to identify the possible genetic underpinnings highlighting these differences in rhabdomyolysis risk across statins. Results: Rhabdomyolysis is a commonly reported adverse event for patients treated with statins, particularly those prescribed simvastatin. Simvastatin was associated with a more than 2-fold increased likelihood of rhabdomyolysis compared to other statins. Men were twice as likely to report rhabdomyolysis than women regardless of statin treatment, with the highest risk observed for pravastatin (ROR = 2.30, *p* < 0.001) and atorvastatin (ROR = 2.03, *p* < 0.0001). Several possible DDIs were identified, including furosemide/Lasix, allopurinol clopidogrel/Plavix, and pantoprazole, which may elevate rhabdomyolysis risk through impaired muscle function and delayed statin metabolism. Finally, nine myopathic genes were identified as possible regulators of statin-induced rhabdomyolysis, including *DYSF*, *DES*, *PLEC*, *CAPN3*, *SCN4A*, *TNNT1*, *SDHA*, *MYH7*, and *PYGM* in primary human muscle cells. Conclusions: Simvastatin was associated with the highest risk of rhabdomyolysis. The risk of rhabdomyolysis was more pronounced in men than women. Several possible DDIs were identified including furosemide/Lasix, allopurinol clopidogrel/Plavix, and pantoprazole.

## 1. Introduction

Rhabdomyolysis is a degradative condition in which there is rapid necrosis of damaged skeletal muscle tissue and a sudden release of various intracellular components, including myoglobin, creatine kinase (CK), aldolase, and electrolytes, into the systemic blood circulation [1,2,3]. An estimated 85% of all rhabdomyolysis results from traumatic injury to muscle tissue caused by crushing mechanical forces while approximately 15% are of nontraumatic origin, such as due to alcohol or drug abuse, side effects of certain medications, prolonged immobilization or hospitalization, various bacterial and viral infections, and certain metabolic disorders [1,2]. Although some patients may present asymptomatically with rhabdomyolysis in mild cases, common reported symptoms include muscle weakness, malaise, dark urine due to myoglobinuria, swelling of the extremities, decreased mobility, and possible acute kidney injury (AKI) [3,4]. The most sensitive tests to detect the presence of skeletal muscle injury and diagnose rhabdomyolysis include serum blood testing for elevated CK levels and urine testing for elevated myoglobin levels [3,5]. Treatment of rhabdomyolysis primarily includes IV fluid and electrolyte infusions to prevent dehydration and the development or progression of AKI as well as physical therapy to restore muscle function and integrity [1,3,6].

Statins are a class of well-known drugs used in the treatment of hypercholesterolemia with the first statin drug, lovastatin (brand name Mevacor), receiving marketing approval in 1987 by the United States (US) Food and Drug Administration (FDA) [7,8,9]. They are among the most prescribed medications globally, with estimated annual sales between 2002 and 2018 of 21.35 million units or USD 24.5 billion [7,10]. According to the Medical Expenditure Panel Survey, a comprehensive survey study conducted annually by the Agency for Healthcare Research and Quality branch of the Department of Health and Human Services in the US to track patient medical expenditures, atorvastatin (brand name Lipitor) was the most prescribed drug in the United States (*n* = 109,582,746) with other statin drugs rosuvastatin (brand name Crestor) (*n* = 37,095,971) and simvastatin (brand name Zocor) (*n* = 26,459,069) being ranked 13th and 19th, respectively [11]. A retrospective cross-sectional study of noninstitutionalized US citizens aged 40 and older (*n* = 409,804) using data derived from MEPS from 2008–2019 found that 22% of surveyed patients were on an active statin regimen and that the total number of statin-treated patients increased from 37 million to 92 million between 2013 and 2019 [12]. Furthermore, among those currently taking an active statin therapy, 36% of patients were being treated with atorvastatin and 34% were being treated with simvastatin [12]. Thus, widespread use and extensive distribution of the statin drug class warrants a comprehensive understanding of their safety profiles. 

Statins function as a competitive inhibitor of hydroxymethylglutaryl-CoA (HMG-CoA) reductase, a key regulator of hepatic cholesterol and triglyceride production [10,13]. By reducing the conversion of HMG-CoA to mevalonic acid and promoting the expression of low-density lipoprotein cholesterol (LDL-C) receptors to expel excess cholesterol from the body, the amount of circulating plasma cholesterol decreases [10,13]. Furthermore, statins shift the equilibrium of cholesterol production in favor of high-density lipoprotein cholesterol (HDL-C) as well as delay arterial plaque accumulation, reducing inflammation, and increasing myocardial blood flow [10,13]. Statins may be further classified based on one of several criteria, including their potency with regards to cholesterol reduction, their solubility, and whether they are natural or synthetic in nature [10,14,15,16]. Low-intensity statins, including fluvastatin (20–40 mg dose), pitavastatin (1 mg), pravastatin (10–20 mg), lovastatin (20 mg), and simvastatin (10 mg), are capable of reducing LDL-C levels by less than 30% while moderate-intensity statins such as fluvastatin (80 mg), pitavastatin (2–4 mg), atorvastatin (10–20 mg), simvastatin (20–40 mg), pravastatin (40–80 mg), lovastatin (40 mg), and rosuvastatin (5–10 mg) are used to reduce LDL-C levels by up to 50% [13,17]. High-intensity statins, which are used to treat patients with significant hypercholesterolemia and reduce LDL-C levels by >50%, includes atorvastatin (40–80 mg) and rosuvastatin (20–40 mg) [13,17]. When classified by solubility, pravastatin and rosuvastatin are considered hydrophilic statins while lipophilic statins, which demonstrate greater extrahepatic properties, includes atorvastatin, lovastatin, simvastatin, fluvastatin, and pitavastatin [13,14]. Finally, synthetic statins, sometimes referred to as super-statins, such as atorvastatin, fluvastatin, pitavastatin, and rosuvastatin typically exhibit stronger cholesterol-reducing capabilities compared to naturally derived statins, including lovastatin and pravastatin [13,18]. Thus, due to the significant pharmacokinetic and pharmacodynamic diversity of statin drugs, it is important to differentiate between their safety profiles to maximize patient safety and minimize adverse events.

Multiple studies have characterized and defined an association between statin therapy and the risk of rhabdomyolysis, a rare but potentially fatal adverse drug event (ADE) [19,20,21]. For instance, a comparative analysis published in the British Journal of Clinical Pharmacology, which assessed 10,657 case reports from the World Health Organization’s VigiBase from 1995–2020, found that the use of any statin was associated with a nearly 60-fold increase (ROR = 59.33, 95% CI 57.88–60.82) in rhabdomyolysis compared to patients not treated with a statin regimen [22]. In particular, simvastatin was associated with a nearly 2-fold increase in rhabdomyolysis (ROR = 2.20, 95% CI 2.11–2.29) compared to those taking another statin drug [22]. A systematic review of data derived from 12 randomized controlled trials indexed in PubMed found that despite low incidence, the use of statin therapy was associated with the development of rhabdomyolysis, particularly at high doses, if simvastatin or rosuvastatin was the prescribed statin therapy, or when statins were used concurrently with other drugs [23]. Concurrent use of certain antibiotics such as daptomycin and clarithromycin, as well as some antifungal medications including itraconazole and clotrimazole, have demonstrated elevated risk of rhabdomyolysis due to adverse drug-drug interactions (DDIs) [20,24]. For instance, a disproportionality analysis of 971,861 adverse event reports dated from the 1st quarter of 2004 to the 4th quarter of 2022 collected from the Food and Drug Administration (FDA) Adverse Event Reporting System (FAERS) found three significant DDIs between the co-administration of daptomycin and a statin drug, including with rosuvastatin (ROR 124.39, 95% CI 87.35–178.47), simvastatin (ROR 94.83, 95% CI 71.12–126.46), and atorvastatin (ROR 68.53, 95% CI 51.93–90.43) [25]. Thus, there is significant evidence in the literature of the potential association between statin therapy and rhabdomyolysis.

Our study will implement a multidisciplinary approach utilizing FAERS and transcriptomic data to further characterize the association between statin therapy and rhabdomyolysis risk as well as providing an explanation as to the genetic components that underpins this association. The effects of certain relevant confounders such as patient gender and age on the strength of the association between statin usage and rhabdomyolysis risk were also investigated. Furthermore, association rule mining was utilized to identify possible DDIs that significantly alter the risk of statin-associated rhabdomyolysis.

## 2. Materials and Methods

### 2.1. FAERS Data

Adverse event reports derived from FAERS were accessed using the publicly available FAERS dashboard tool. Each record derived from FAERS contains the following seven distinct data entries:

(1) Drug information
(2) Drug-related adverse events (ADEs)
(3) All reported patient outcomes for the ADE indicated
(4) Demographic information including patient sex, age, and body weight
(5) Source that reported the ADE
(6) Date of submission for the ADE report
(7) The indications for use of each indicated drug

To ensure the reliability of the conclusions, only statins with case numbers more than 5000 were included. Consequently, this paper focuses on five statins: atorvastatin, simvastatin, lovastatin, rosuvastatin, and pravastatin.

Reports were extracted from the FAERS dashboard using both the generic drug term (atorvastatin, simvastatin, lovastatin, rosuvastatin, and pravastatin) as well as brand names for each drug (Lipitor, Zocor, Mevacor, Crestor, and Pravachol) as input search terms. Any report in which the generic or brand name of a drug of interest is indicated in the ‘suspect product names’ or ‘suspect product active ingredients’ data columns will subsequently be outputted and downloaded for analysis.

### 2.2. Removal of Duplicate FAERS Reports

Most of the approximately 30 million records accessible through the FAERS dashboard are reports submitted directly to the FDA by a patient, health provider, pharmaceutical company, or drug manufacturer. However, a subset of these reports may instead be indirectly reported from publications in the literature. Consequently, there is a risk of duplicate reports in which multiple records, each of which with different case IDs, are included in the FAERS dashboard despite pertaining to the same patient or case. As a result, any records derived from publications were ultimately excluded from analysis for three reasons. First, these indirectly reported records may be submitted by multiple sources such as different drug manufacturers or clinicians, resulting in some cases in which records are duplicated 2–10 times for the same patient and same adverse event [26]. If these duplicate reports were included in the final analysis, the strength of the possible safety signal may be inflated and there is a greater risk of identifying false positive safety signals. Next, there may be inconsistencies between the reports submitted by different reporters, with certain information emphasized or removed entirely based on varying interpretations of the data or differing study goals. Finally, as these records only comprise a small portion of all records contained in the FAERS dashboard (approximately 2–3%), exclusion of these reports would ultimately have a negligible effect on any conclusions drawn from this study. Data analysis was performed using R statistical software version 4.3.2.

### 2.3. Disproportionality Analysis

A disproportionality analysis was conducted to compare the risk of a rhabdomyolysis adverse event between patients prescribed one of five statins: a lipophilic statin, including atorvastatin, simvastatin or lovastatin, or a hydrophilic statin, including rosuvastatin or pravastatin. The odds ratio and the corresponding 95% confidence interval (CI) for these comparisons were then calculated. A significant safety signal was defined by any association measure in which the lower bound of the corresponding 95% CI was greater than 1.0, indicating a significantly greater likelihood of reporting rhabdomyolysis in the treatment group compared to the reference group (simvastatin in this case). Odds ratios for each association were calculated using the Medcalc odds ratio online calculator available at https://www.medcalc.org/calc/odds_ratio.php (accessed on 1 December 2024).

### 2.4. Association Rule Mining (ARM)

Finally, association rule mining (ARM) was used to identify other ADEs that commonly presented alongside statin-induced rhabdomyolysis for each of the five statins assessed in this study. Each association rule was analyzed using the arules R package. The following formulas were used to determine the support, confidence, and lift values for each association rule:

(1) $$Support\left(\left\{X\right\}\to \left\{Y\right\}\right)=\frac{Number\;of\;reports\;containing\;X\;and\;Y}{Total\;number\;of\;reports}$$

(2) $$Lift\left(\left\{X\right\}\to \left\{Y\right\}\right)=\frac{Support\left(X\to Y\right)}{Support\left(X\right)\times Support(Y)}$$

(3) $$Confidence\left(\left\{X\right\}\to \left\{Y\right\}\right)=\frac{Number\;of\;reports\;containing\;X\;and\;Y}{Number\;of\;reports\;containing\;X}$$

### 2.5. Transcriptomic Data Analysis

A public transcriptomic dataset from the NCBI GEO database (GEO id: GSE107998) was analyzed to explore the genes in human muscle cells regulated by statin drugs [27]. In this dataset, primary human muscle cells were treated by simvastatin (a lipophilic statin) and rosuvastatin (a hydrophilic statin). Briefly, primary human myoblasts were cultured in ProVitro skeletal muscle growth medium. Treatment groups were treated with either 5 μM simvastatin or 5 μM rosuvastatin dissolved in DMSO and compared to two different control groups, including untreated cells and cells treated with 7 μM DMSO. Each control and treatment group had 4 replicates with treatment added 24 hrs after cells were plated. The gene expression of these cells with and without statins were evaluated by RNA-seq. Differentially expressed genes were identified using the limma package in R. This list of differentially expressed genes was then used as an input to identify enriched disease pathways using the ShinyGo V0.81 web tool.

## 3. Results

### 3.1. Top 10 ADEs of Each Statin

First, the top 10 most frequently reported ADEs were identified for the following 5 statin drugs: rosuvastatin, pravastatin, simvastatin, atorvastatin, and rosuvastatin. Myalgia was the most frequently reported adverse event for all five statin drugs. Of the 51,446 reports indicating simvastatin use, the remaining most reported adverse events included rhabdomyolysis (*n* = 6145), drug interaction (*n* = 4874), fatigue (*n* = 3292), arthralgia (*n* = 3141), elevated blood creatinine phosphokinase (*n* = 3051), muscular weakness (*n* = 3007), asthenia (*n* = 2865), pain in extremity (*n* = 2722), and nausea (*n* = 2480). Of the 101,774 reports indicating atorvastatin use, the remaining most reported adverse events included type II diabetes (*n* = 10,397), arthralgia (*n* = 4780), pain in extremity (*n* = 4714), fatigue (*n* = 4373), muscle spasms (*n* = 4227), asthenia (*n* = 4136), rhabdomyolysis (*n* = 4068), muscular weakness (*n* = 3880), and drug interaction (*n* = 3814).

Of the 55,489 reports indicating rosuvastatin use, the remaining most commonly reported adverse events included pain in extremity (*n* = 3057), rhabdomyolysis (*n* = 2879), muscle spasms (*n* = 2820), fatigue (*n* = 2764), pain (*n* = 2762), arthralgia (*n* = 2685), dyspnea (*n* = 2346), asthenia (*n* = 2295), and product dose omission issue (*n* = 2146). 

Of the 15,701 reports indicating lovastatin use, the remaining most reported adverse events including elevated blood creatine phosphokinase levels (*n* = 1316), abnormal hepatic function (*n* = 992), drug ineffective (*n* = 961), asthenia (*n* = 598), alopecia (*n* = 567), pain (*n* = 542), hypercholesterolemia (*n* = 533), arthralgia (*n* = 516), and myopathy (*n* = 496).

**Figure 1:**
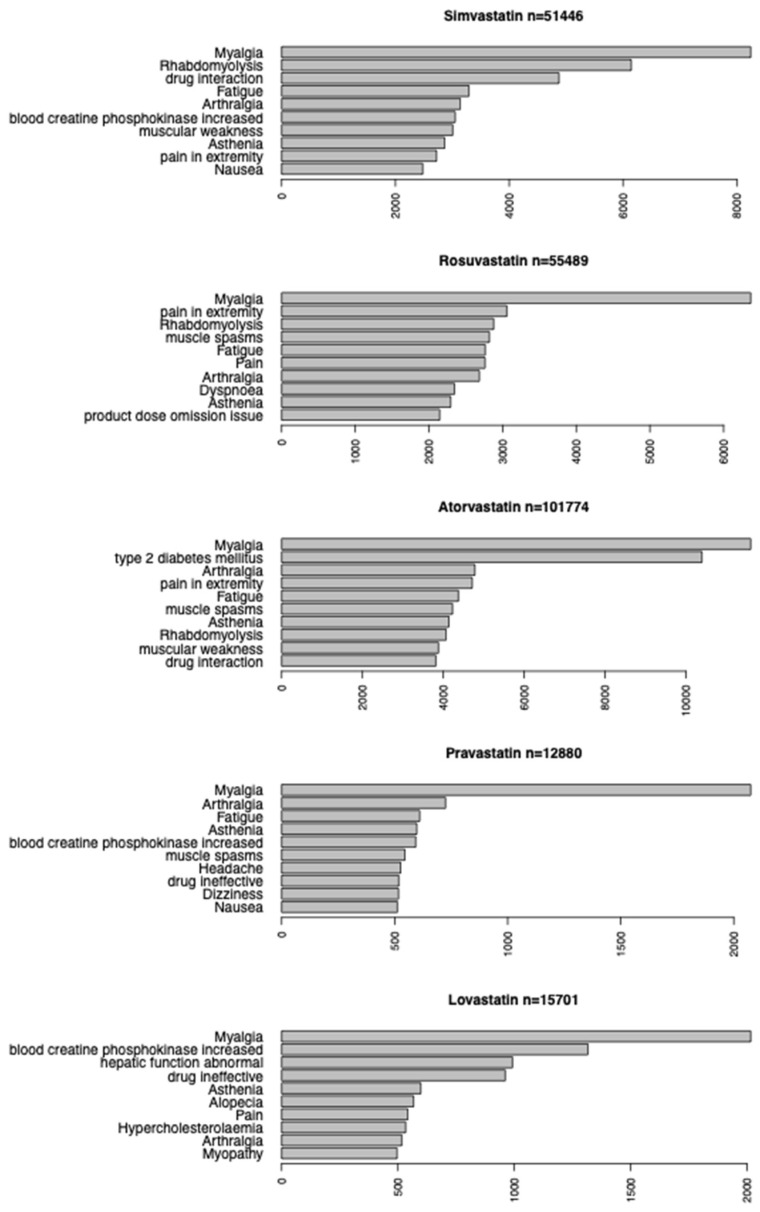
Top 10 reported adverse events for 5 statin drugs.

Finally, of the 12,880 reports indicating pravastatin use, the remaining most reported adverse events included arthralgia (*n* = 725), fatigue (*n* = 611), asthenia (*n* = 598), elevated blood creatine phosphokinase levels (*n* = 593), muscle spasms (*n* = 545), headache (*n* = 526), drug ineffective (*n* = 518), dizziness (*n* = 517), and nausea (*n* = 512). Rhabdomyolysis was identified as the 2nd, 8th, and 3rd most reported ADE for simvastatin, atorvastatin, and rosuvastatin, respectively, while it was ranked 26th and 23rd, respectively, for lovastatin and pravastatin, indicating greater risk of drug-associated rhabdomyolysis. These results are summarized in Figure 1.

### 3.2. ADES Associated with Rhabdomyolysis

**Figure 2:**
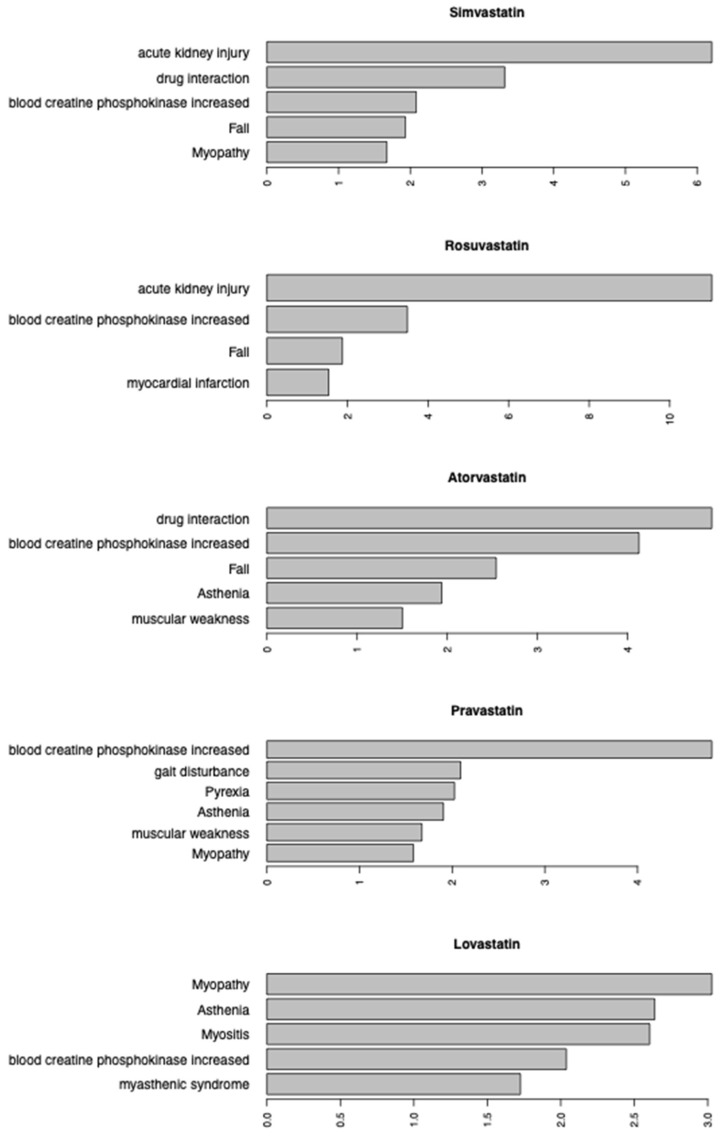
Association rule mining of ADEs associated with Rhabdomyolysis for five statins (lift cutoff value = 1.5).

Next, association rule mining was used to identify additional ADEs that frequently co-presented alongside rhabdomyolysis in individuals treated with a statin therapy. A lift value greater than 1 indicates that rhabdomyolysis and a given ADE were indicated together more frequently than the expected number of instances. A lift criterion of ≥1.5 was used as the cutoff to identify significant rhabdomyolysis-ADE pairs in those treated with atorvastatin, rosuvastatin, simvastatin, lovastatin, or pravastatin, with the top five significant ADE pairs presented in Figure 2.

For those treated with atorvastatin, drug interactions (lift = 4.93), elevated blood creatine phosphokinase levels (lift = 4.12), fall (lift = 2.54), asthenia (lift = 1.94), and muscular weakness (lift =1.50) were ADEs that commonly presented alongside rhabdomyolysis. Individuals that were treated with rosuvastatin and reported rhabdomyolysis also frequently reported acute kidney injury (lift = 11.04), elevated blood creatine phosphokinase levels (lift = 3.49), fall (lift = 1.87), and myocardial infarction (lift = 1.53), while rhabdomyolysis frequently co-presented with acute kidney injury (lift = 6.20), drug interactions (lift = 3.32), elevated blood creatine phosphokinase levels (lift = 2.08), fall (lift = 1.93), and myopathy (lift = 1.67).

Individuals that were treated with lovastatin and reported rhabdomyolysis frequently reported myopathy (lift = 3.03), asthenia (lift = 2.64), myositis (lift = 2.60), elevated blood creatine phosphokinase levels (lift = 2.04), and myasthenic syndrome (lift = 1.72) as concurrent ADEs. Finally, rhabdomyolysis frequently co-presented with elevated blood creatine phosphokinase levels (lift = 4.80), gait disturbance (lift = 2.09), pyrexia (lift = 2.02), asthenia (lift = 1.90), muscular weakness (lift = 1.67), and myopathy (lift = 1.58) in those treated with pravastatin.

Overall, elevated blood creatine phosphokinase levels, an indicator of kidney injury, was commonly reported alongside rhabdomyolysis regardless of which statin was used as therapy. Furthermore, asthenia was a commonly co-presenting ADE in those treated with atorvastatin, lovastatin, or pravastatin, while drug interactions (atorvastatin and simvastatin), acute kidney injury (atorvastatin and simvastatin), and fall (atorvastatin and rosuvastatin) were frequently reported as co-presenting ADEs for two different statins.

### 3.3. Comparison of Rhabdomyolysis Risk Across Statins

Next, the likelihood of reporting rhabdomyolysis as an ADE was compared between each statin drug. As shown in Table 1, compared to those treated with simvastatin, the risk of reporting rhabdomyolysis was lower in each of the other statin drugs analyzed in this study. Individuals treated with rosuvastatin had an approximately 62% lower likelihood (OR = 0.38, *p*-value < 0.0001) of reporting rhabdomyolysis as an ADE while treatment with atorvastatin was associated with a 69% reduction (OR = 0.31, *p*-value < 0.0001) in rhabdomyolysis risk. In addition, utilizing lovastatin or pravastatin was associated with an 82% lower (OR = 0.18, *p*-value < 0.0001) and 78% lower (OR = 0.22, *p*-value < 0.0001) likelihood of reporting rhabdomyolysis as an ADE, respectively, when compared to those treated with simvastatin.

### 3.4. Rhabdomyolysis Risk for Single Drug Use

In the previous section, all reports indicating the use of a statin drug were analyzed, regardless of whether or not the use of additional therapeutics were indicated. To isolate the effects of a particular statin drug on rhabdomyolysis risk and mitigate the potential effects of other drugs that are taken concurrently, only reports indicating statin therapy exclusively were considered in this section. As shown in Table 2, simvastatin was associated with the highest risk of rhabdomyolysis. Comparatively, individuals that were treated with rosuvastatin had an approximately 54% lower likelihood (OR = 0.46, *p* < 0.0001) of rhabdomyolysis development while those treated with atorvastatin was associated with a 73% reduction in rhabdomyolysis risk (OR = 0.27, *p*-value < 0.0001) compared to those administered simvastatin. Similarly, individuals treated with pravastatin demonstrated a 79% lower likelihood (OR = 0.21, *p*-value < 0.0001) of rhabdomyolysis compared to those administered simvastatin for treatment. Finally, lovastatin was associated with the lowest likelihood of reporting rhabdomyolysis as an ADE with a 90% reduction in rhabdomyolysis risk (OR = 0.10, *p*-value < 0.0001) compared to those on a simvastatin regimen.

### 3.5. Sex Differences in Rhabdomyolysis Risk

The potential effects of patient gender as a possible confounder were considered in the association between rhabdomyolysis risk and statin therapy for each of the five statins assessed in this study. As shown in Table 3, males had a comparatively higher risk of rhabdomyolysis than females administered the same statin therapy for all five statins. Males treated with simvastatin had a 1.71-fold increase (OR = 1.71, *p*-value < 0.0001) in rhabdomyolysis risk compared to females while treatment with rosuvastatin was associated with a nearly 2-fold increase (OR = 1.74, *p*-value < 0.0001) in the likelihood of rhabdomyolysis development for males compared to females. Males treated with atorvastatin had a 102% elevation (OR = 2.02, *p*-value < 0.0001) in rhabdomyolysis risk compared to females while males treated with pravastatin had a 2.30-fold increase (OR = 2.30, *p*-value < 0.0001) in the likelihood of rhabdomyolysis development compared to females. Finally, there was a 40% increase (OR = 1.40, *p*-value < 0.0001) in rhabdomyolysis risk in males treated with lovastatin compared to females administered the same statin therapy.

### 3.6. Possible DDIs with Statins

**Figure 3:**
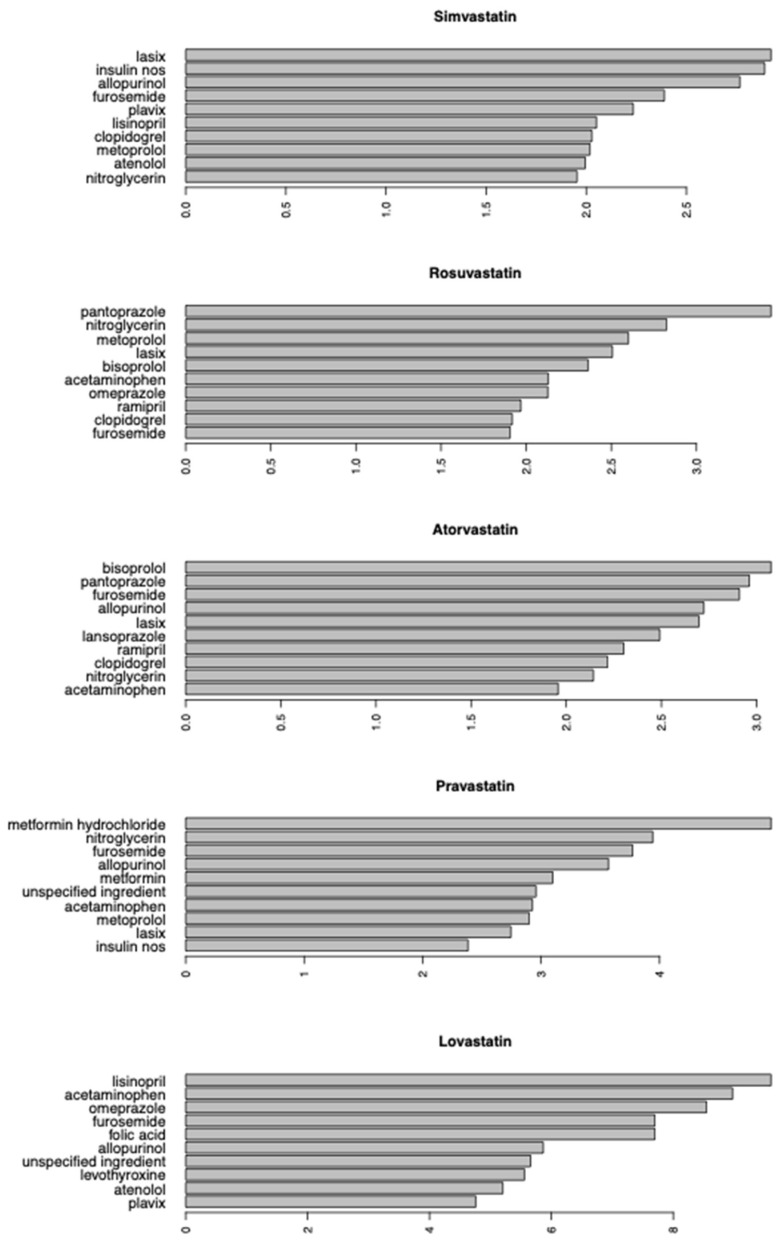
Association rule mining of DDIs associated with elevated rhabdomyolysis risk for five statins (lift cutoff value = 1.5).

Next, association rule mining was used to identify possible DDIs between a single statin therapy and another drug that were associated with elevated rhabdomyolysis risk (Figure 3). For simvastatin, the top 10 DDIs most frequently associated with rhabdomyolysis was Lasix (lift = 2.92), insulin nos (lift = 2.89), allopurinol (lift = 2.77), furosemide (lift = 2.39), Plavix (lift = 2.23), lisinopril (lift = 2.05), clopidogrel (lift = 2.03), metoprolol (lift = 2.02), atenolol (lift = 1.99), and nitroglycerin (lift = 1.95). For rosuvastatin, the top 10 DDIs most frequently associated with rhabdomyolysis was pantoprazole (lift = 3.44), nitroglycerin (lift = 2.82), metoprolol (lift = 2.60), Lasix (lift = 2.51), bisoprolol (lift = 2.36), acetaminophen (lift = 2.13), omeprazole (lift = 2.13), ramipril (lift = 1.97), clopidogrel (lift = 1.92), and furosemide (lift = 1.90). For atorvastatin, the top 10 DDIs most frequently associated with rhabdomyolysis was bisoprolol (lift = 3.08), pantoprazole (lift = 2.96), furosemide (lift = 2.91), allopurinol (lift = 2.72), Lasix (lift = 2.70), lansoprazole (lift = 2.49), ramipril (lift = 2.30), clopidogrel (lift = 2.22), nitroglycerin (lift = 2.14), and acetaminophen (lift = 1.96). For pravastatin, the top 10 DDIs most frequently associated with rhabdomyolysis was metformin hydrochloride (lift = 4.94), nitroglycerin (lift = 3.94), furosemide (lift = 3.77), allopurinol (lift = 3.57), metformin (lift = 3.10), unspecified ingredient (lift = 2.96), acetaminophen (lift = 2.93), metoprolol (lift = 2.90), Lasix (lift = 2.74), and insulin nos (lift = 2.38). Finally, for lovastatin, the top 10 DDIs most frequently associated with rhabdomyolysis was lisinopril (lift = 9.60), acetaminophen (lift = 8.97), omeprazole (lift = 8.54), furosemide (lift = 7.70), folic acid (lift = 7.70), allopurinol (lift = 5.86), unspecified ingredient (lift = 5.66), levothyroxine (lift = 5.56), atenolol (lift = 5.20), and Plavix (lift = 4.76).

Furosemide/Lasix were the most common drugs indicated as possibly interacting with a statin drug and were ranked 4th/1st, 4th/10th, 3rd/5th, 3rd/9th, and 4th for simvastatin, rosuvastatin, atorvastatin, pravastatin, and lovastatin, respectively. Furthermore, allopurinol comprised the top 10 for DDIs for 4 of the statins analyzed, including atorvastatin (4th), simvastatin (3rd), lovastatin (6th), and pravastatin (4th). In addition, nitroglycerin was indicated as a possible DDI with 4 statins, including atorvastatin (9th), rosuvastatin (2nd), simvastatin (10th), and pravastatin (2nd). Next, clopidogrel/Plavix was ranked 7th/5th, 9th, 8th, and 10th for simvastatin, rosuvastatin, atorvastatin, and lovastatin, respectively. Finally, pantoprazole was identified as a possible DDI with both atorvastatin (2nd) and rosuvastatin (1st).

### 3.7. Genes Regulated by Human Muscle Cells

**Figure 4:**
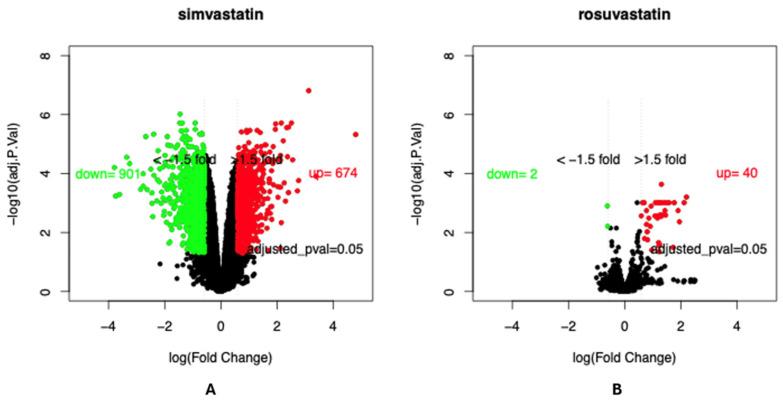
Differentially expressed genes for simvastatin-treated primary muscle cells (**A**) and rosuvastatin-treated primary muscle cells (**B**) compared to DMSO-treated control cells. In both cases, significantly downregulated genes are indicated in green while significantly upregulated genes are indicated in red.

Finally, transcriptomic analysis of a public RNA-seq dataset GEO ID: GSE107998 was performed to identify key target genes that are differentially expressed across treatment groups and that may drive the progression of statin-induced rhabdomyolysis. In this dataset, primary muscle cells were treated with either simvastatin (lipophilic) or rosuvastatin (hydrophilic). Differentially expressed genes were identified using an adjusted *p*-value < 0.05 and a log fold change ≥1.5 for upregulated genes or log fold change ≤−1.5 for downregulated genes. As shown in Figure 4, when compared to control cells that were only treated with DMSO, simvastatin-treated muscle cells resulted in a total of 1575 differentially expressed genes, 901 of which were upregulated and 674 of which were downregulated. Comparatively, rosuvastatin-treated cells gave rise to 44 differentially expressed genes, 42 of which were upregulated and 2 of which were downregulated. The significantly larger genetic perturbation observed in simvastatin-treated primary muscle cells may explain the elevated risk of rhabdomyolysis in those treated with simvastatin and, possibly by extension, other lipophilic statins compared to those treated with rosuvastatin or other hydrophilic statins.

### 3.8. Disease Pathways Enriched in Simvastatin-Treated Muscle Cells

Next, the list of differentially expressed genes served as an input for the ShinyGO V0.81 enrichment tool in order to identify disease and molecular pathways that were enriched in simvastatin-treated muscle cells compared to those treated with rosuvastatin. Comparatively, the only Online Mendelian Inheritance in Man (OMIM) disease pathway upregulated in simvastatin-treated muscle cells was myopathy, a general term for muscle weakness or dysfunction, with nine unique genes mapped to this pathway, including *PGAM2*, *NEB*, *DES*, *TNNT1*, *CRYAB*, *MYH7*, *MYF6*, *DYSF*, and *ATP2A1*. When mapping to the Disease Ontology, three muscle-related diseases were enriched including myoglobinuria (*LPIN1*, *PGAM2*, and *GSR*), muscular disease (*DES*, *CLU*, *PRKCD*, *HMGCR*, *CAPN3*, *SDHA*, *PPARA*, and *PYGM*), and myopathy (*DYSF*, *DES*, *PLEC*, *CAPN3*, *SCN4A*, *TNNT1*, *SDHA*, *MYH7*, and *PYGM*) (Table 4). The genes *DES*, *TNNT1*, *MYH7*, and *DYSF* were identified as important regulatory genes in both the OMIM and KEGG myopathy pathway.

## 4. Discussion

In this study, a multidisciplinary approach was utilized to compare the risk of rhabdomyolysis across different statin drugs as well as identify possible regulatory genes that modulate this differential risk. Furthermore, we wanted to identify additional adverse events that are frequently co-presenting in patients that report suspected statin-induced rhabdomyolysis. Rhabdomyolysis, a severe form of muscle dysfunction characterized by significant muscle breakdown, was most commonly reported by those prescribed a lipophilic statin, including simvastatin and atorvastatin, compared to those treated with a hydrophilic statin, which is in agreement in present literature [22,28,29]. Unlike hydrophilic statins, which demonstrate greater hepatic selectivity than lipophilic statins and require specific protein carriers that are primarily found on hepatic cells to penetrate tissues, lipophilic statins can more easily passively diffuse into non-hepatic cells, including muscle fibers, and accumulate [29,30,31]. This accumulation would thus lead to greater targeted cell membrane disruption of muscle cells due to depleted cholesterol levels as well as due to decreased production of ubiquinone, an important antioxidant and a component of the electron transport chain [32,33,34,35]. In several studies, ubiquinone deficiency has been linked to myopathy and muscle damage, while ubiquinone supplementation has demonstrated efficacy in restoring proper muscle function [32,33,36]. Therefore, these myotoxic effects may be more pronounced in those treated with a lipophilic statin, particularly at high doses or those already presenting with general muscle dysfunction.

The difference in the observed likelihood of rhabdomyolysis in this study may be a consequence of several factors. First, lovastatin, the first naturally derived statin approved for use by the FDA in 1987, has the lowest bioavailability among commonly prescribed statin drugs at 5% (compared to 12% and 20% for atorvastatin and rosuvastatin, respectively) [37,38,39]. Although simvastatin has similarly poor oral bioavailability, simvastatin exhibits significantly more lipophilic properties and is usually prescribed at significantly higher doses than lovastatin, which is classified as a low-intensity statin [17,39]. Because of these properties, simvastatin is more likely to confer off-target effects than lovastatin, such as greater muscle instability and injury [29,40]. Like lovastatin, pravastatin is a largely hydrophilic statin that has greater hepatic specificity and greater excretion through the kidneys, thus being less likely to accumulate in muscle tissue and elicit adverse muscle-related side effects [17,39]. However, despite having significantly greater oral bioavailability compared to lovastatin (18% vs. 5%), pravastatin has approximately 50% lower binding affinity for HMG-CoA reductase, consequently mitigating the potency of the drug and the possibility of myopathy [39,41]. Finally, pravastatin is a not a prodrug and does not undergo further metabolic breakdown by CYP enzymes into other active metabolites, thus reducing the risk of myopathies related to possible statin DDIs [10,39,42].

In the absence of statin therapy, men may be at greater risk of rhabdomyolysis than women of comparable age due to higher muscle mass and a greater risk of muscle injury [43,44]. Furthermore, men are more likely to engage in highly strenuous physical activity, particularly while experiencing bacterial or viral infections, and experience exercise-related dehydration and electrolyte imbalances, further elevating rhabdomyolysis risk in men [3,45]. In addition, men are more likely to engage in other modifiable risk factors such as drug and alcohol abuse, as well as use creatine supplements that increase muscle stress and drive elevated rhabdomyolysis risk [3,46]. Finally, men are more susceptible and more likely to inherit genetic drivers of rhabdomyolysis, including mitochondrial myopathies, fatty acid metabolism disorders such as carnitine palmitoyltransferase and very-long-chain acyl-CoA dehydrogenase deficiencies, polymorphisms in muscle metabolism genes such as α-actinin-3, or certain x-linked glycogen storage diseases (GSDs), including GSD type IX and phosphoglycerate kinase 1 deficiency [43,47,48]. Thus, men may have a greater baseline risk of rhabdomyolysis than women.

Compared to women, men may also be more susceptible to statin-related toxicity than women. For instance, men may metabolize certain statins that are metabolized by cytochrome p450 3A4 (CYP3A4), such as simvastatin, atorvastatin, and lovastatin, at a slower rate compared to women due to lower CYP3A4 activity in men, resulting in decreased clearance of the drug and greater potential for adverse DDIs due to sustained systemic bioavailability [34,40,49]. For other statins that are not metabolized by cytochrome p450 enzymes, such as pravastatin, the observed elevated risk of rhabdomyolysis in men may be due to differences in fat deposition and liver function. For instance, overweight and obese men tend to have a greater concentration of abdominal visceral fat than women, which is more greatly associated with decreased metabolism and liver function than the accumulation of subcutaneous fat observed in many women [50,51]. Furthermore, men are more at risk of liver damage not only due to modifiable habits such as binge drinking but also due to the lack of the hepatoprotective effects provided by estrogen [52,53]. Thus, men may have greater difficulty in the clearance of pravastatin, which is primarily metabolized in the stomach and excreted in the liver, resulting in statin-related toxicity [54].

Other genetic differences may explain, at least in part, the elevated risk of rhabdomyolysis in men compared to women. Organic anion transporting polypeptide 1B1 (OATP1B1) is a membrane bound protein highly expressed in the liver and is the primary driver of statin clearance in the liver while organic anion transporter 3 (OAT3) is predominantly responsible for the absorption and clearance of statins by the kidney [55,56,57]. Rat models have demonstrated elevated expression of both OATP1B1 and OAT3 expression in female-derived liver and kidney tissue, respectively, compared to male rats, indicating possible elevated statin clearance rates in females compared to males for hydrophilic statins that are more significantly excreted by the kidneys, in the case of OAT3, and lipophilic statins that are primarily excreted by the liver, in the case of OATP1B1 [58,59]. Thus, males may be at greater risk of toxicity related to statin accumulation and rhabdomyolysis due to slower excretion rates of statins from the body.

In addition to differences in expression levels of transport proteins, polymorphisms in the genes encoding for these transporters have also demonstrated significant effects on statin excretion and the risk of statin-induced rhabdomyolysis. For instance, one study found that single nucleotide polymorphisms such as c.521T > C in the *SLCO1B1* gene, which encodes OATP1B1, were associated with a 2-fold increase in risk of all myopathies and was also shown to be significantly associated with simvastatin-induced myopathies [60]. Other studies have corroborated the association of different OATP1B1 mutations including c.521T > C with reduced statin excretion and elevated rhabdomyolysis risk [55,61,62,63,64]. Thus, mutant alleles in some patients may contribute to the elevated risk of statin-induced myopathies due to impaired statin clearance and increased systemic toxicity due to higher circulating levels of statin compounds.

While statin therapy demonstrates efficacy in both men and women, studies have shown that women are both less likely to be prescribed statin therapy and are also more likely to deny statin therapy recommendations if provided. For instance, a study published in the Journal of the American Heart Association that analyzed data of 5693 participants collected from the Patient and Provider Assessment of Lipid Management Registry found that women were 30% less likely to be prescribed a statin therapy compared to men when adjusting for various confounders including age, income levels, education status, patient beliefs, and provider characteristics (adjusted OR = 0.70, 95% CI 0.61–0.81) [65]. The same study also demonstrated that women were more likely to reject statin therapy (3.6% vs. 2.0%, *p* < 0.001) as well as were more likely to discontinue statin therapy (10.9% vs. 6.1%, *p* < 0.001) [65]. A retrospective cohort study (*n* = 24,212) of patients at high risk of cardiovascular disease and were not taking an active statin therapy found that women were 18% less likely to accept a statin therapy recommendation (OR = 0.82, 95% CI 0.78–0.88) than men when accounting for demographic and socioeconomic covariates and were 50% more likely to never initiate statin therapy over the course of the study [66]. Thus, the differential risk of reporting rhabdomyolysis as an ADE based on sex may be attributed, at least in part, to differences in prescribing frequency, therapy acceptance, and length of treatment window.

Several drugs were determined to be strong candidates for possible DDIs with concurrent statin therapy, including furosemide/Lasix, allopurinol, nitroglycerin, clopidogrel/Plavix, and pantoprazole. Furosemide is a loop diuretic that inhibits the reabsorption of sodium by the kidneys, resulting in increased secretion of water and potassium in the urine [67,68]. In some instances, furosemide can induce severe electrolyte imbalance and hypokalemia in the blood, which may restrict blood flow to various muscle groups, decrease muscle contraction, promote muscle ischemia, and drive further muscle damage [68,69,70]. This may synergistically drive rhabdomyolysis risk alongside concurrent statin therapy, particularly lipophilic statins that are more readily absorbed by muscle cells and potentially more myotoxic than hydrophilic statins, by compounding muscle injury and exacerbating hypokalemic-induced muscle paralysis [34,71]. In particular, the upregulation of SCN4A, a voltage-gated sodium channel, in simvastatin-treated cells may strengthen the inward-facing sodium current and exacerbate decreased serum potassium levels, thus increasing hypokalemic periodic muscle paralysis and rhabdomyolysis risk [72,73].

The effects of allopurinol on rhabdomyolysis and related muscle injuries in the literature are mixed. Some studies demonstrate the protective effect of allopurinol on exercise-induced AKI and other muscle injuries [74,75,76]. Conversely, hypouricemia, a possible side effect of allopurinol treatment if uric acid levels decline too much, may actually increase the risk of exercise-induced AKI and drive further kidney injury in those already presenting with rhabdomyolysis [77,78]. Although no-specific simvastatin-allopurinol interactions are indicated in the literature, allopurinol, particularly at high doses or those with pre-existing kidney conditions, may indirectly elevate the risk of rhabdomyolysis and severe muscle injury by increasing potassium excretion by the kidneys and driving hypokalemia progression [79,80].

Clopidogrel/Plavix is a blood thinner prodrug medication used to lower the risk of stroke, blood clots, and cardiovascular events by inhibiting platelet aggregation [81]. Individually, clopidogrel may elevate rhabdomyolysis risk by the over suppression of platelet aggregation, which is required for proper skeletal muscle recovery and regeneration from injury [82]. Furthermore, clopidogrel has been linked to rhabdomyolysis and worsening of kidney function in some patients [83,84]. Concurrent use of the blood thinner clopidogrel and a statin may drive rhabdomyolysis development through 1 of 2 mechanisms. Some studies indicate that statins metabolized by CYP3A4 may serve as competitive inhibitors of the enzyme, thus reducing the efficacy of clopidogrel, promoting platelet activation, and increasing muscle injury due to increased oxidative stress in the first mechanism [85,86,87,88,89]. Conversely, other studies have demonstrated that clopidogrel may instead serve as a competitive inhibitor of statin activity by delaying statin metabolism and clearance, resulting in elevated circulating levels of the statin drug and increased risk of muscle injury or rhabdomyolysis [90,91,92]. An observational study performed using adverse event reports collected from the pharmacovigilance database VigiBase (*n* = 2464) found that a combination therapy of a related antiplatelet medication ticagrelor and either rosuvastatin or atorvastatin was associated with a 90% increase (ROR = 1.90, 95% CI 1.42–2.54) and 30% increase (ROR = 1.30, 95% CI 1.02–1.65), respectively, in rhabdomyolysis risk [92]. Another review of the literature also indicated a strong correlation in rhabdomyolysis risk related to the interaction between statin therapy and antiplatelet activity due to ticagrelor, with emphasis placed on impaired statin metabolism by CYP3A4 [90]. Thus, there is precedence in the literature to indicate that the interaction between statins and antiplatelet drugs such as clopidogrel may elevate the risk of rhabdomyolysis in some patients. 

Finally, pantoprazole is a proton-pump inhibitor used to reduce the production of stomach acid [93]. In general, proton-pump inhibitors such as pantoprazole are directly associated with rhabdomyolysis risk when used individually [94,95,96]. However, interactions between atorvastatin or simvastatin with pantoprazole are well-characterized and are thought to drive elevated rhabdomyolysis risk by function as a CYP3A4 inhibitor and impairing the metabolic breakdown of the statin drug [97,98]. Several studies have demonstrated the efficacy of both pantoprazole and omeprazole in the inhibition of CYP3A4-mediated metabolic breakdown of multiple drugs [99,100,101]. With regards to rosuvastatin, which is not metabolized by CYP3A4 and exhibits greater excretion by the kidneys, pantoprazole, omeprazole, and other proton-pump inhibitors competitively inhibit OAT3 activity and prolong elevated serum concentration, resulting in increased risk of statin-induced rhabdomyolysis [102,103,104]. 

Several genes were found to be significantly enriched by simvastatin treatment in primary human muscle cells and were mapped to both OMIM as well as KEGG pathways for myopathy, including *DES*, *TNNT1*, *MYH7*, and *DYSF*. *DES* (Desmin) encodes a crucial protein in the structural integrity and function of skeletal muscles and its upregulation is associated with various myopathies associated with muscle weakness and rhabdomyolysis [105,106]. *TNNT1* (Troponin T1, Slow Skeletal Type) encodes a protein that regulates the contraction of slow twitch striated muscles and is released from injured muscle tissue, thus serving as a diagnostic marker of exercise-induced muscle injury and possible rhabdomyolysis [107,108,109]. *MYH7* (Myosin Heavy Chain 7) encodes the β isoform of the myosin heavy chain that is commonly found in slow twitch skeletal muscles, mutations of which result in various conditions with known elevated rhabdomyolysis risk under physical stress including hypertrophic cardiomyopathy and myosin storage myopathy [110,111]. The upregulation of *DYSF* (Dysferlin) has previously been shown to be associated with muscle weakness and irregular gait while mutations in this gene have been linked to various myopathies, including limb-girdle muscular dystrophy type 2B and Miyoshi myopathy [112,113]. Gain-of-function mutations or upregulation of *PYGM* (Muscle Glycogen Phosphorylase) and *PGAM2* (Phosphoglycerate Mutase 2) give rise to glycogen storage diseases, resulting in severe muscle weakness, exercise intolerance, and an increased risk of muscle injury [114,115,116]. Finally, mutations in metabolic genes such as *SDHA* (Succinate Dehydrogenase Complex Flavoprotein Subunit A) and *LPIN1* (Lipin 1) are associated with impaired lipid metabolism in muscle cells as well as increased muscle breakdown and elevated mitochondrial dysfunction [117,118,119]. Thus, simvastatin may elevate rhabdomyolysis risk through impaired skeletal muscle integrity, altered metabolism, and greater susceptibility to muscle injury due to weakness.

Despite these promising findings, the study presents some limitations. First, the inherent constraints of the observational study design only allow for determination of an association between statin therapy and elevated rhabdomyolysis risk. Consequently, a causal relationship between these two variables cannot be ascertained. Additionally, the FAERS dashboard report does not provide dosage information, limiting the ability to assess the impact of dosage on the association between statins and the risk of rhabdomyolysis. Furthermore, the proposed DDIs identified also only establish an association between the two drugs, and further clinical studies are needed to verify the accuracy and severity of these indicated interactions. In addition, further in vivo and in vitro studies are needed to validate the genes postulated to be key regulators of statin-induced rhabdomyolysis risk. These differentially expressed genes may not fully extrapolate to other cell types and these regulators may not be universal across multiple cell types. Data concerning the length of overlap time period between a suspected statin-drug DDI were unavailable and the dose of statins used in the RNA seq analysis may be higher than the mean serum concentration in humans. For instance, the mean serum concentration of simvastatin ranges from 2.2–4.3 nM/L while the primary muscle cells were treated with 5 μM [120]. Thus, other regulatory genes may also be involved in the development of statin-induced rhabdomyolysis in humans treated at more clinically relevant statin doses. Nonetheless, this study provides further insight into the differential risk of rhabdomyolysis risk across statin therapies and enhances the possibility of personalized medicine based on an individual’s pre-existing muscle injury risk. Further studies will explore the genetic underpinnings of statin-induced rhabdomyolysis for other statin drugs to further characterize this potentially severe adverse event.

## 5. Conclusions

Rhabdomyolysis is a commonly reported adverse event for patients treated with statins, particularly those prescribed simvastatin, atorvastatin, or rosuvastatin. Other commonly reported adverse events alongside rhabdomyolysis including myopathy, AKI, fall, disturbed gait, and elevated blood creatine phosphokinase levels. Simvastatin was associated with a more than 2-fold increased likelihood of rhabdomyolysis compared to other statins and men were twice as likely to report rhabdomyolysis than women regardless of statin treatment. Several possible DDIs were identified, including furosemide/Lasix, allopurinol clopidogrel/Plavix, and pantoprazole, which may elevate rhabdomyolysis risk due to development of hypokalemic conditions, impaired muscle function, and delayed statin metabolism. Finally, nine myopathic genes were identified as possible regulators of statin-induced rhabdomyolysis, including *DYSF*, *DES*, *PLEC*, *CAPN3*, *SCN4A*, *TNNT1*, *SDHA*, *MYH7*, and *PYGM.*

## Figures and Tables

**Table 1:** Comparison of rhabdomyolysis risk across five statins.

Statins	With ADE	Without ADE	Odds Ratio (*p*-Value)
Simvastatin (Zocor, Flolipid)	5030	46,416	1
Rosuvastatin (Crestor, Ezallor)	2217	53,272	0.3840 (*p* < 0.0001)
Atorvastatin (Lipitor)	3306	98,468	0.3098 (*p* < 0.0001)
Pravastatin (Pravachol)	297	12,583	0.2178 (*p* < 0.0001)
Lovastatin (Altoprev)	300	15,401	0.1798 (*p* < 0.0001)

**Table 2:** Comparison of rhabdomyolysis risk across five statins in patients only treated with a single drug.

Statins	With ADE	Without ADE	Odds Ratio (*p*-Value)
Simvastatin (Zocor, Flolipid)	962	11,252	1
Rosuvastatin (Crestor, Ezallor)	699	17,893	0.4569 (*p* < 0.0001)
Atorvastatin (Lipitor)	822	35,980	0.2672 (*p* < 0.0001)
Pravastatin (Pravachol)	65	3618	0.2101 (*p* < 0.0001)
Lovastatin (Altoprev)	64	7720	0.0970 (*p* < 0.0001)

**Table 3:** Sex differences in rhabdomyolysis across five statins.

Statins	Gender	With ADE	Without ADE	Odds Ratio (*p*-Value)
Simvastatin (Zocor, Flolipid)	Female	1669	20,693	
	Male	3034	21,941	1.7145 (*p* < 0.0001)
Rosuvastatin (Crestor, Ezallor)	Female	828	27,691	
	Male	1138	21,896	1.7381 (*p* < 0.0001)
Atorvastatin (Lipitor)	Female	1096	47,460	
	Male	1851	39,628	2.0226 (*p* < 0.0001)
Pravastatin (Pravachol)	Female	91	5860	
	Male	179	5003	2.3040 (*p* < 0.0001)
Lovastatin (Altoprev)	Female	119	6677	
	Male	155	6186	1.4059 (*p* < 0.0001)

**Table 4:** Diseases enriched in the genes up-regulated by Simvastatin.

Diseases	Num of Genes	Pathway Genes	Fold Enrichment	Enrichment FDR	Gene List
OMIM ^1^ disease					
Myopathy	9	36	9.09	1.6 × 10^−5^	PGAM2 NEB DES TNNT1 CRYAB MYH7 MYF6 DYSF ATP2A1
Disease Ontology					
DOID:0080108 myoglobinuria	3	4	27.28	0.0062	LPIN1 PGAM2 GSR
DOID:11100 Q fever	4	6	24.25	0.0011	DHCR24 HMGCR LDLR LSS
DOID:9455 lipid storage disease	7	21	12.13	0.00026	DEPP1 WIPI1 FADS2 SCD LSS NUPR1 INHBE
DOID:423 myopathy	9	48	6.82	0.00086	DYSF DES PLEC CAPN3 SCN4A TNNT1 SDHA MYH7 PYGM
DOID:397 restrictive cardiomyopathy	7	38	6.70	0.0057	SYNE2 DES TRPM4 PDLIM3 PRKCD TNNT1 MYH7
DOID:0080000 muscular disease	8	44	6.61	0.0024	DES CLU PRKCD HMGCR CAPN3 SDHA PPARA PYGM
DOID:9000808 Hypercholesterolemia	12	71	6.15	0.00026	CYP51A1 ACAT2 CSF1 HMGCR GSR NR4A1 VLDLR LDLR MVD LSS SQLE ITIH4

^1^ OMIM: Online Mendelian Inheritance in Man.

## Data Availability

The original contributions presented in this study are included in the article. Further inquiries can be directed to the corresponding author.

## Abbreviations

The following abbreviations are used in this manuscript:

CK	Creatine kinase
AKI	Acute kidney injury
DDI	Drug-drug interaction
FDA	Food and Drug Administration
FAERS	FDA Adverse Event Reporting System
ADE	Adverse drug event
HMG-CoA	Hydroxymethylglutaryl-CoA
ROR	Reporting odds ratio
CI	Confidence interval
ARM	Association rule mining
CYP3A4	Cytochrome p450 3A4
GSD	Glycogen storage disease
OMIM	Online Mendelian Inheritance in Man
KEGG	Kyoto Encyclopedia of Genes and Genomes
OATP1B1	Organic anion transporting polypeptide 1B1
OAT3	Organic anion transporter 3
DYSF	Dysferlin
DES	Desmin
PLEC	Plectin
CAPN3	Calpain 3
SCN4A	Sodium Channel, Voltage-Gated, Type IV α Subunit
TNNT1	Troponin T1, Slow Skeletal Type
SDHA	Succinate Dehydrogenase Complex Flavoprotein Subunit A
MYH7	Myosin Heavy Chain 7
PYGM	Muscle Glycogen Phosphorylase
LPIN1	Lipin 1
PGAM2	Phosphoglycerate mutase

## References

[1] Stanley M., Chippa V., Aeddula N.R., Quintanilla Rodriguez B.S., Adigun R. (2024). Rhabdomyolysis. StatPearls.

[2] Cabral B.M.I., Edding S.N., Portocarrero J.P., Lerma E.V. (2020). Rhabdomyolysis. Dis. Mon..

[3] Torres P.A., Helmstetter J.A., Kaye A.M., Kaye A.D. (2015). Rhabdomyolysis: Pathogenesis, Diagnosis, and Treatment. Ochsner J..

[4] Stahl K., Rastelli E., Schoser B. (2020). A Systematic Review on the Definition of Rhabdomyolysis. J. Neurol..

[5] Torr L., Mortimore G. (2022). The Management and Diagnosis of Rhabdomyolysis-Induced Acute Kidney Injury: A Case Study. Br. J. Nurs..

[6] Parekh R., Care D.A., Tainter C.R. (2012). Rhabdomyolysis: Advances in Diagnosis and Treatment. Emerg. Med. Pract..

[7] Lin S.Y., Baumann K., Zhou C., Zhou W., Cuellar A.E., Xue H. (2021). Trends in Use and Expenditures for Brand-Name Statins after Introduction of Generic Statins in the US, 2002–2018. JAMA Netw. Open.

[8] Endo A. (2010). A Historical Perspective on the Discovery of Statins. Proc. Jpn. Acad. Ser. B Phys. Biol. Sci..

[9] Chester A., El Guindy A. (2021). From Fleming to Endo: The Discovery of Statins. Glob. Cardiol. Sci. Pract..

[10] Sizar O., Khare S., Patel P., Talati R. (2024). Statin Medications. StatPearls.

[11] Cohen J.W., Monheit A.C., Beauregard K.M., Cohen S.B., Lefkowitz D.C., Potter D.E., Sommers J.P., Taylor A.K., Arnett R.H. (1996). The Medical Expenditure Panel Survey: A National Health Information Resource. Inquiry.

[12] Matyori A., Brown C.P., Ali A., Sherbeny F. (2023). Statins Utilization Trends and Expenditures in the U.S. Before and after the Implementation of the 2013 ACC/AHA Guidelines. Saudi Pharm. J..

[13] Bansal A.B., Cassagnol M. (2024). HMG-CoA Reductase Inhibitors. StatPearls.

[14] Climent E., Benaiges D., Pedro-Botet J. (2021). Hydrophilic or Lipophilic Statins?. Front. Cardiovasc. Med..

[15] Dormuth C.R., Hemmelgarn B.R., Paterson J.M., James M.T., Teare G.F., Raymond C.B., Lafrance J.P., Levy A., Garg A.X., Ernst P. (2013). Use of High Potency Statins and Rates of Admission for Acute Kidney Injury: Multicenter, Retrospective Observational Analysis of Administrative Databases. BMJ.

[16] Khatiwada N., Hong Z. (2024). Potential Benefits and Risks Associated with the Use of Statins. Pharmaceutics.

[17] Kim J., Park K.T., Jang M.J., Park T.K., Lee J.M., Yang J.H., Song Y.B., Choi S.H., Gwon H.C., Lee S.H. (2018). High-Intensity Versus Non-High-Intensity Statins in Patients Achieving Low-Density Lipoprotein Cholesterol Goal after Percutaneous Coronary Intervention. J. Am. Heart Assoc..

[18] Sadowska A., Osinski P., Roztocka A., Kaczmarz-Chojnacka K., Zapora E., Sawicka D., Car H. (2023). Statins-from fungi to Pharmacy. Int. J. Mol. Sci..

[19] Kumar S., Anne S., B H.K. (2021). Statin Induced Rhabdomyolysis. J. Assoc. Physicians India.

[20] Ezad S., Cheema H., Collins N. (2018). Statin-Induced Rhabdomyolysis: A Complication of a Commonly Overlooked Drug Interaction. Oxf. Med. Case Rep..

[21] Simons J.E., Holbrook A.M., Don-Wauchope A.C. (2015). Successful Reintroduction of Statin Therapy after Statin-Associated Rhabdomyolysis. J. Clin. Lipidol..

[22] Montastruc J.L. (2023). Rhabdomyolysis and Statins: A Pharmacovigilance Comparative Study between Statins. Br. J. Clin. Pharmacol..

[23] Safitri N., Alaina M.F., Pitaloka D.A.E., Abdulah R. (2021). A Narrative Review of Statin-Induced Rhabdomyolysis: Molecular Mechanism, Risk Factors, and Management. Drug Healthc. Patient Saf..

[24] Dybro A.M., Damkier P., Rasmussen T.B., Hellfritzsch M. (2016). Statin-Associated Rhabdomyolysis Triggered by Drug-Drug Interaction with Itraconazole. BMJ Case Rep..

[25] Wei C., Yin W., He Z., Wu B. (2023). Reporting of Drug-Induced Myopathies Associated with the Combination of Statins and Daptomycin: A Disproportionality Analysis Using the US Food and Drug Administration Adverse Event Reporting System. J. Clin. Med..

[26] Han W., Morris R., Bu K., Zhu T., Huang H., Cheng F. (2024). Analysis of Literature-Derived Duplicate Records in the FDA Adverse Event Reporting System (FAERS) Database. Can. J. Physiol. Pharmacol..

[27] Grunwald S.A., Popp O., Haafke S., Jedraszczak N., Grieben U., Saar K., Patone G., Kress W., Steinhagen-Thiessen E., Dittmar G. (2020). Statin-Induced Myopathic Changes in Primary Human Muscle Cells and Reversal by a Prostaglandin F2 Alpha Analogue. Sci. Rep..

[28] Di Stasi S.L., MacLeod T.D., Winters J.D., Binder-Macleod S.A. (2010). Effects of Statins on Skeletal Muscle: A Perspective for Physical Therapists. Phys. Ther..

[29] Vinci P., Panizon E., Tosoni L.M., Cerrato C., Pellicori F., Mearelli F., Biasinutto C., Fiotti N., Di Girolamo F.G., Biolo G. (2021). Statin-Associated Myopathy: Emphasis on Mechanisms and Targeted Therapy. Int. J. Mol. Sci..

[30] Zhang X., Lou D., Fu R., Wu F., Zheng D., Ma X. (2024). Association between Statins Types with Incidence of Liver Cancer: An Updated Meta-Analysis. Curr. Med. Chem..

[31] Fong C.W. (2014). Statins in Therapy: Understanding Their Hydrophilicity, Lipophilicity, Binding to 3-Hydroxy-3-Methylglutaryl-CoA Reductase, Ability to Cross the Blood Brain Barrier and Metabolic Stability Based on Electrostatic Molecular Orbital Studies. Eur. J. Med. Chem..

[32] Muraki A., Miyashita K., Mitsuishi M., Tamaki M., Tanaka K., Itoh H. (2012). Coenzyme Q10 Reverses Mitochondrial Dysfunction in Atorvastatin-Treated Mice and Increases Exercise Endurance. J. Appl. Physiol..

[33] Skarlovnik A., Janic M., Lunder M., Turk M., Sabovic M. (2014). Coenzyme Q10 Supplementation Decreases Statin-Related mild-to-Moderate Muscle Symptoms: A Randomized Clinical Study. Med. Sci. Monit..

[34] Ward N.C., Watts G.F., Eckel R.H. (2019). Statin Toxicity. Circ. Res..

[35] Choi H.K., Won E.K., Choung S.Y. (2016). Effect of Coenzyme Q10 Supplementation in Statin-Treated Obese Rats. Biomol. Ther..

[36] Horvath R., Schneiderat P., Schoser B.G., Gempel K., Neuen-Jacob E., Ploger H., Muller-Hocker J., Pongratz D.E., Naini A., DiMauro S. (2006). Coenzyme Q10 Deficiency and Isolated Myopathy. Neurology.

[37] Li J., Di L., Cheng X., Ji W., Piao H., Cheng G., Zou M. (2020). The Characteristics and Mechanism of Co-Administration of Lovastatin Solid Dispersion with Kaempferol to Increase Oral Bioavailability. Xenobiotica.

[38] Zhou J., Zhou D. (2015). Improvement of Oral Bioavailability of Lovastatin by Using Nanostructured Lipid Carriers. Drug Des. Dev. Ther..

[39] Schachter M. (2005). Chemical, Pharmacokinetic and Pharmacodynamic Properties of Statins: An Update. Fundam. Clin. Pharmacol..

[40] Jeeyavudeen M.S., Pappachan J.M., Arunagirinathan G. (2022). Statin-Related Muscle Toxicity: An Evidence-Based Review. Touchreviews Endocrinol..

[41] Murphy C., Deplazes E., Cranfield C.G., Garcia A. (2020). The Role of Structure and Biophysical Properties in the Pleiotropic Effects of Statins. Int. J. Mol. Sci..

[42] Jacobson T.A. (2004). Comparative Pharmacokinetic Interaction Profiles of Pravastatin, Simvastatin, and Atorvastatin When Coadministered with Cytochrome P450 Inhibitors. Am. J. Cardiol..

[43] Rawson E.S., Clarkson P.M., Tarnopolsky M.A. (2017). Perspectives on Exertional Rhabdomyolysis. Sports Med..

[44] Burgess S. (2022). Rhabdomyolysis: An Evidence-Based Approach. J. Intensive Care Soc..

[45] Carneiro A., Viana-Gomes D., Macedo-da-Silva J., Lima G.H.O., Mitri S., Alves S.R., Kolliari-Turner A., Zanoteli E., Neto F.R.A., Palmisano G. (2021). Risk Factors and Future Directions for Preventing and Diagnosing Exertional Rhabdomyolysis. Neuromuscul. Disord..

[46] Kim J., Lee J., Kim S., Ryu H.Y., Cha K.S., Sung D.J. (2016). Exercise-Induced Rhabdomyolysis Mechanisms and Prevention: A Literature Review. J. Sport Health Sci..

[47] Wieser T., Adam M.P., Feldman J., Mirzaa G.M., Pagon R.A., Wallace S.E., Amemiya A. (1993). Carnitine Palmitoyltransferase II Deficiency. GeneReviews^®^.

[48] Scalco R.S., Gardiner A.R., Pitceathly R.D., Zanoteli E., Becker J., Holton J.L., Houlden H., Jungbluth H., Quinlivan R. (2015). Rhabdomyolysis: A Genetic Perspective. Orphanet J. Rare Dis..

[49] Yoon S., Jeong S., Jung E., Kim K.S., Jeon I., Lee Y., Cho J.Y., Oh W.Y., Chung J.Y. (2021). Effect of CYP3A4 Metabolism on Sex Differences in the Pharmacokinetics and Pharmacodynamics of Zolpidem. Sci. Rep..

[50] Yang X., Sui W., Zhang M., Dong M., Lim S., Seki T., Guo Z., Fischer C., Lu H., Zhang C. (2017). Switching Harmful Visceral Fat to Beneficial Energy Combustion Improves Metabolic Dysfunctions. JCI Insight.

[51] Nauli A.M., Matin S. (2019). Why Do Men Accumulate Abdominal Visceral Fat?. Front. Physiol..

[52] Ezhilarasan D. (2020). Critical Role of Estrogen in the Progression of Chronic Liver Diseases. Hepatobiliary Pancreat. Dis. Int..

[53] Palmisano B.T., Zhu L., Stafford J.M. (2017). Role of Estrogens in the Regulation of Liver Lipid Metabolism. Adv. Exp. Med. Biol..

[54] Hatanaka T. (2000). Clinical Pharmacokinetics of Pravastatin: Mechanisms of Pharmacokinetic Events. Clin. Pharmacokinet..

[55] Ciuta A.D., Nosol K., Kowal J., Mukherjee S., Ramirez A.S., Stieger B., Kossiakoff A.A., Locher K.P. (2023). Structure of Human Drug Transporters OATP1B1 and OATP1B3. Nat. Commun..

[56] Windass A.S., Lowes S., Wang Y., Brown C.D. (2007). The Contribution of Organic Anion Transporters OAT1 and OAT3 to the Renal Uptake of Rosuvastatin. J. Pharmacol. Exp. Ther..

[57] Kalliokoski A., Niemi M. (2009). Impact of OATP Transporters on Pharmacokinetics. Br. J. Pharmacol..

[58] Breljak D., Brzica H., Sweet D.H., Anzai N., Sabolic I. (2013). Sex-Dependent Expression of Oat3 (Slc22a8) and Oat1 (Slc22a6) Proteins in Murine Kidneys. Am. J. Physiol. Renal Physiol..

[59] Taniguchi T., Zanetti-Yabur A., Wang P., Usyk M., Burk R.D., Wolkoff A.W. (2020). Interindividual Diversity in Expression of Organic Anion Uptake Transporters in Normal and Cirrhotic Human Liver. Hepatol. Commun..

[60] Carr D.F., O’Meara H., Jorgensen A.L., Campbell J., Hobbs M., McCann G., van Staa T., Pirmohamed M. (2013). SLCO1B1 Genetic Variant Associated with Statin-Induced Myopathy: A Proof-of-Concept Study Using the Clinical Practice Research Datalink. Clin. Pharmacol. Ther..

[61] Lee H.H., Ho R.H. (2017). Interindividual and Interethnic Variability in Drug Disposition: Polymorphisms in Organic Anion Transporting Polypeptide 1B1 (OATP1B1; SLCO1B1). Br. J. Clin. Pharmacol..

[62] Tamraz B., Fukushima H., Wolfe A.R., Kaspera R., Totah R.A., Floyd J.S., Ma B., Chu C., Marciante K.D., Heckbert S.R. (2013). OATP1B1-Related Drug-Drug and Drug-Gene Interactions as Potential Risk Factors for Cerivastatin-Induced Rhabdomyolysis. Pharmacogenet. Genom..

[63] Canestaro W.J., Austin M.A., Thummel K.E. (2014). Genetic Factors Affecting Statin Concentrations and Subsequent Myopathy: A HuGENet Systematic Review. Genet. Med..

[64] Turner R.M., Pirmohamed M. (2019). Statin-Related Myotoxicity: A Comprehensive Review of Pharmacokinetic, Pharmacogenomic and Muscle Components. J. Clin. Med..

[65] Nanna M.G., Wang T.Y., Xiang Q., Goldberg A.C., Robinson J.G., Roger V.L., Virani S.S., Wilson P.W.F., Louie M.J., Koren A. (2019). Sex Differences in the Use of Statins in Community Practice. Circ. Cardiovasc. Qual. Outcomes.

[66] Brown C.J., Chang L.S., Hosomura N., Malmasi S., Morrison F., Shubina M., Lan Z., Turchin A. (2023). Assessment of Sex Disparities in Nonacceptance of Statin Therapy and Low-Density Lipoprotein Cholesterol Levels Among Patients at High Cardiovascular Risk. JAMA Netw. Open.

[67] Ruisz W., Stollberger C., Finsterer J., Weidinger F. (2013). Furosemide-Induced Severe Hypokalemia with Rhabdomyolysis without Cardiac Arrest. BMC Womens Health.

[68] Lin Z., Wong L.Y.F., Cheung B.M.Y. (2022). Diuretic-Induced Hypokalaemia: An Updated Review. Postgrad. Med. J..

[69] Ozgur B., Kursat S. (2002). Hypokalemic Rhabdomyolysis Aggravated by Diuretics Complicating Conn’s Syndrome without Acute Renal Failure. Clin. Nephrol..

[70] Lindinger M.I., Cairns S.P. (2021). Regulation of Muscle Potassium: Exercise Performance, Fatigue and Health Implications. Eur. J. Appl. Physiol..

[71] Wang S., Ran Y., Chen X., Li C., Cheng S., Liu J. (2020). Pleiotropic Effects of Simvastatin on the Regulation of Potassium Channels in Monocytes. Front. Pharmacol..

[72] Hu N.Q., Yang J.Y., Lv J.L., Zhu Y.Z., Li L.H. (2024). Hypokalemic Periodic Paralysis Type 2 Due to SCN4A Val1105Met Mutation: A Case Study. Cureus.

[73] Shibano M., Kubota T., Kokubun N., Miyaji Y., Kuriki H., Ito Y., Hamanoue H., Takahashi M.P. (2022). Periodic Paralysis due to Cumulative Effects of Rare Variants in SCN4A with Small Functional Alterations. Muscle Nerve.

[74] Gois P.H.F., Canale D., Volpini R.A., Ferreira D., Veras M.M., Andrade-Oliveira V., Camara N.O.S., Shimizu M.H.M., Seguro A.C. (2016). Allopurinol Attenuates Rhabdomyolysis-Associated Acute Kidney Injury: Renal and Muscular Protection. Free Radic. Biol. Med..

[75] Sanchis-Gomar F., Pareja-Galeano H., Perez-Quilis C., Santos-Lozano A., Fiuza-Luces C., Garatachea N., Lippi G., Lucia A. (2015). Effects of Allopurinol on Exercise-Induced Muscle Damage: New Therapeutic Approaches?. Cell Stress Chaperones.

[76] Ferrando B., Gomez-Cabrera M.C., Salvador-Pascual A., Puchades C., Derbre F., Gratas-Delamarche A., Laparre L., Olaso-Gonzalez G., Cerda M., Viosca E. (2018). Allopurinol Partially Prevents Disuse Muscle Atrophy in Mice and Humans. Sci. Rep..

[77] Otani N., Ouchi M., Misawa K., Hisatome I., Anzai N. (2022). Hypouricemia and Urate Transporters. Biomedicines.

[78] Hosoyamada M. (2021). Hypothetical Mechanism of Exercise-Induced Acute Kidney Injury Associated with Renal Hypouricemia. Biomedicines.

[79] Hestin D., Johns E.J. (1999). The Influence of Allopurinol on Kidney Haemodynamic and Excretory Responses to Renal Ischaemia in Anaesthetized Rats. Br. J. Pharmacol..

[80] De Becker B., Hupkens E., Dewachter L., Coremans C., Delporte C., van Antwerpen P., Franck T., Zouaoui Boudjeltia K., Cullus P., van de Borne P. (2021). Acute Effects of Hypouricemia on Endothelium, Oxidative Stress, and Arterial Stiffness: A Randomized, Double-Blind, Crossover Study. Physiol. Rep..

[81] Beavers C.J., Naqvi I.A. (2024). Clopidogrel. StatPearls.

[82] Graca F.A., Stephan A., Minden-Birkenmaier B.A., Shirinifard A., Wang Y.D., Demontis F., Labelle M. (2023). Platelet-Derived Chemokines Promote Skeletal Muscle Regeneration by Guiding Neutrophil Recruitment to Injured Muscles. Nat. Commun..

[83] Burton J.R., Burton I., Pearson G.J. (2007). Clopidogrel-Precipitated Rhabdomyolysis in a Stable Heart Transplant Patient. Ann. Pharmacother..

[84] Ram R., Swarnalatha G., Ramesh V., Rao K.N., Dakshinamurty K.V. (2013). Rhabdomyolysis Induced Acute Renal Failure Secondary to Statins. Indian J. Nephrol..

[85] Tirkkonen T., Heikkila P., Vahlberg T., Huupponen R., Laine K. (2013). Epidemiology of CYP3A4-Mediated Clopidogrel Drug-Drug Interactions and Their Clinical Consequences. Cardiovasc. Ther..

[86] Clarke T.A., Waskell L.A. (2003). The Metabolism of Clopidogrel is Catalyzed by Human Cytochrome P450 3A and Is Inhibited by Atorvastatin. Drug Metab. Dispos..

[87] D’Amico A., Cavarretta E., Fossati C., Borrione P., Pigozzi F., Frati G., Sciarretta S., Costa V., De Grandis F., Nigro A. (2022). Platelet Activation Favours NOX2-Mediated Muscle Damage in Elite Athletes: The Role of Cocoa-Derived Polyphenols. Nutrients.

[88] Suarez Ferreira S.P., Hall R.P., Majumdar M., Goudot G., Jessula S., Bellomo T., Lee I., Kukreja N., Parmar G., Boada A.E. (2023). Atorvastatin Effect on Clopidogrel Efficacy in Patients with Peripheral Artery Disease. Ann. Vasc. Surg..

[89] Leoncini M., Toso A., Maioli M., Bellandi F. (2013). Statin and Clopidogrel Pharmacological Interaction. G. Ital. Cardiol..

[90] Danielak D., Karazniewicz-Lada M., Glowka F. (2018). Assessment of the Risk of Rhabdomyolysis and Myopathy During Concomitant Treatment with Ticagrelor and Statins. Drugs.

[91] Wang Z.Y., Chen M., Zhu L.L., Yu L.S., Zeng S., Xiang M.X., Zhou Q. (2015). Pharmacokinetic Drug Interactions with Clopidogrel: Updated Review and Risk Management in Combination Therapy. Ther. Clin. Risk Manag..

[92] Roule V., Alexandre J., Lemaitre A., Chretien B., Sassier M., Fedrizzi S., Beygui F., Dolladille C. (2024). Rhabdomyolysis with Co-Administration of Statins and Antiplatelet Therapies-Analysis of the WHO Pharmacovigilance Database. Cardiovasc. Drugs Ther..

[93] Bernshteyn M.A., Masood U. (2024). Pantoprazole. StatPearls.

[94] Duncan S.J., Howden C.W. (2017). Proton Pump Inhibitors and Risk of Rhabdomyolysis. Drug Saf..

[95] Mitsuboshi S., Hamano H., Kuniki Y., Niimura T., Chuma M., Ushio S., Lin T.J., Matsumoto J., Takeda T., Kajizono M. (2023). Proton Pump Inhibitors and Rhabdomyolysis: Analysis of Two Different Cross-Sectional Databases. Ann. Pharmacother..

[96] Sun Y., Zhang A., Zuo M., Chen J., Zhu L. (2024). A Pharmacovigilance Study of Association between Proton-Pump Inhibitors and Rhabdomyolysis Event Based on FAERS Database. J. Gastroenterol. Hepatol..

[97] Le J., Liao Y., Li S., Chen X., Hong Z. (2021). High-throughput LC-MS/MS Method for Simultaneous Determination of Pantoprazole and Atorvastatin in Rat Plasma: Application to a Pharmacokinetic Interaction Study. Curr. Drug Metab..

[98] Bogman K., Peyer A.K., Torok M., Kusters E., Drewe J. (2001). HMG-CoA Reductase Inhibitors and P-Glycoprotein Modulation. Br. J. Pharmacol..

[99] Shirasaka Y., Sager J.E., Lutz J.D., Davis C., Isoherranen N. (2013). Inhibition of CYP2C19 and CYP3A4 by Omeprazole Metabolites and Their Contribution to Drug-Drug Interactions. Drug Metab. Dispos..

[100] Miedziaszczyk M., Idasiak-Piechocka I. (2023). Safety Analysis of Co-Administering Tacrolimus and Omeprazole in Renal Transplant Recipients—A Review. Biomed. Pharmacother..

[101] Li X.Q., Andersson T.B., Ahlstrom M., Weidolf L. (2004). Comparison of Inhibitory Effects of the Proton Pump-Inhibiting Drugs Omeprazole, Esomeprazole, Lansoprazole, Pantoprazole, and Rabeprazole on Human Cytochrome P450 Activities. Drug Metab. Dispos..

[102] Andre C., Mernissi T., Choukroun G., Bennis Y., Kamel S., Liabeuf S., Bodeau S. (2021). The Prescription of Drugs That Inhibit Organic Anion Transporters 1 or 3 Is Associated with the Plasma Accumulation of Uremic Toxins in Kidney Transplant Recipients. Toxins.

[103] Wang Y., Ren J., Sun Q., Zhang Z., Lin Y., Deng S., Wang C., Huo X., Sun C., Tian X. (2019). Organic Anion Transporter 3 (OAT3)-Mediated Transport of Dicaffeoylquinic Acids and Prediction of Potential Drug-Drug Interaction. Eur. J. Pharm. Sci..

[104] Zhang J., Wang H., Fan Y., Yu Z., You G. (2021). Regulation of Organic Anion Transporters: Role in Physiology, Pathophysiology, and Drug Elimination. Pharmacol. Ther..

[105] van Spaendonck-Zwarts K.Y., van Hessem L., Jongbloed J.D., de Walle H.E., Capetanaki Y., van der Kooi A.J., van Langen I.M., van den Berg M.P., van Tintelen J.P. (2011). Desmin-Related Myopathy. Clin. Genet..

[106] Savarese M., Sarparanta J., Vihola A., Jonson P.H., Johari M., Rusanen S., Hackman P., Udd B. (2020). Panorama of the Distal Myopathies. Acta Myol..

[107] Tanindi A., Cemri M. (2011). Troponin Elevation in Conditions Other Than Acute Coronary Syndromes. Vasc. Health Risk Manag..

[108] Sorichter S., Mair J., Koller A., Gebert W., Rama D., Calzolari C., Artner-Dworzak E., Puschendorf B. (1997). Skeletal Troponin I as a Marker of Exercise-Induced Muscle Damage. J. Appl. Physiol..

[109] Barthel B.L., Cox D., Barbieri M., Ziemba M., Straub V., Hoffman E.P., Russell A.J. (2021). Elevation of Fast but Not Slow Troponin I in the Circulation of Patients with Becker and Duchenne Muscular Dystrophy. Muscle Nerve.

[110] Fiorillo C., Astrea G., Savarese M., Cassandrini D., Brisca G., Trucco F., Pedemonte M., Trovato R., Ruggiero L., Vercelli L. (2016). MYH7-Related Myopathies: Clinical, Histopathological and Imaging Findings in a Cohort of Italian Patients. Orphanet J. Rare Dis..

[111] Gao Y., Peng L., Zhao C. (2024). MYH7 in Cardiomyopathy and Skeletal Muscle Myopathy. Mol. Cell. Biochem..

[112] Glover L.E., Newton K., Krishnan G., Bronson R., Boyle A., Krivickas L.S., Brown R.H. (2010). Dysferlin Overexpression in Skeletal Muscle Produces a Progressive Myopathy. Ann. Neurol..

[113] Millay D.P., Maillet M., Roche J.A., Sargent M.A., McNally E.M., Bloch R.J., Molkentin J.D. (2009). Genetic Manipulation of Dysferlin Expression in Skeletal Muscle: Novel Insights into Muscular Dystrophy. Am. J. Pathol..

[114] Carvalho A.A.S., Christofolini D.M., Perez M.M., Alves B.C.A., Rodart I., Figueiredo F.W.S., Turke K.C., Feder D., Junior M.C.F., Nucci A.M. (2020). PYGM mRNA Expression in McArdle Disease: Demographic, Clinical, Morphological and Genetic Features. PLoS ONE.

[115] Kruijt N., van den Bersselaar L.R., Kamsteeg E.J., Verbeeck W., Snoeck M.M.J., Everaerd D.S., Abdo W.F., Jansen D.R.M., Erasmus C.E., Jungbluth H. (2021). The Etiology of Rhabdomyolysis: An Interaction between Genetic Susceptibility and External Triggers. Eur. J. Neurol..

[116] Scalco R.S., Snoeck M., Quinlivan R., Treves S., Laforet P., Jungbluth H., Voermans N.C. (2016). Exertional Rhabdomyolysis: Physiological Response or Manifestation of an Underlying Myopathy?. BMJ Open Sport Exerc. Med..

[117] Meijer I.A., Sasarman F., Maftei C., Rossignol E., Vanasse M., Major P., Mitchell G.A., Brunel-Guitton C. (2015). LPIN1 Deficiency with Severe Recurrent Rhabdomyolysis and Persistent Elevation of Creatine Kinase Levels due to Chromosome 2 Maternal Isodisomy. Mol. Genet. Metab. Rep..

[118] Zeharia A., Shaag A., Houtkooper R.H., Hindi T., de Lonlay P., Erez G., Hubert L., Saada A., de Keyzer Y., Eshel G. (2008). Mutations in LPIN1 Cause Recurrent Acute Myoglobinuria in Childhood. Am. J. Hum. Genet..

[119] Siebers E.M., Choi M.J., Tinklenberg J.A., Beatka M.J., Ayres S., Meng H., Helbling D.C., Takizawa A., Bennett B., Garces A.M. (2018). Sdha+/− Rats Display Minimal Muscle Pathology Without Significant Behavioral or Biochemical Abnormalities. J. Neuropathol. Exp. Neurol..

[120] Bjorkhem-Bergman L., Lindh J.D., Bergman P. (2011). What Is a Relevant Statin Concentration in Cell Experiments Claiming Pleiotropic Effects?. Br. J. Clin. Pharmacol..

